# Extreme Few-View Tomography without Training Data

**DOI:** 10.26717/bjstr.2024.55.008672

**Published:** 2024-02-23

**Authors:** Gengsheng L Zeng

**Affiliations:** 1 Department of Computer Science, Utah Valley University, USA; 2 Department of Radiology and Imaging Sciences, University of Utah, USA

**Keywords:** Inverse Problem, Optimization, Total-Variation Minimization, Few-View Tomography, Iterative Algorithm, Image Reconstruction

## Abstract

There are fewer than 10 projection views in extreme few-view tomography. The state-of-the-art methods to reconstruct images with few-view data are compressed sensing based. Compressed sensing relies on a sparsification transformation and total variation (TV) norm minimization. However, for the extreme few-view tomography, the compressed sensing methods are not powerful enough. This paper seeks additional information as extra constraints so that extreme few-view tomography becomes possible. In transmission tomography, we roughly know the linear attenuation coefficients of the objects to be imaged. We can use these values as extra constraints. Computer simulations show that these extra constraints are helpful and improve the reconstruction quality.

## Introduction

XTREME few-view tomography is referred to the situation where the number of tomography measurement views is less than 10 [1–3]. If we model the data acquisition as a system of linear equations, the system is extremely under- determined for extreme few-view tomography. Constraints are vital in shrinking the solution space [4]. Iterative algorithms are better than analytical algorithms when the imaging system is under-determined [5–9]. The total-variation (TV) norm of the gradient of an image is a good indicator for the piecewise-constant feature of the image. TV minimization is a popular method for few-view tomography [10–15]. Extreme few-view tomography requires more information about the target image in addition to the piecewise-constant constraint. In the era of machine learning, a large amount of information can be learned from images like the image to be reconstructed [16–22]. This paper assumes that similar images are not available. We must seek other information. In transmission tomography, we roughly know the values of the attenuation coefficients for the materials in the objects being imaged. We use these known values as the constraints in this paper, as described in the next section.

## Methods

**Figure 1:**
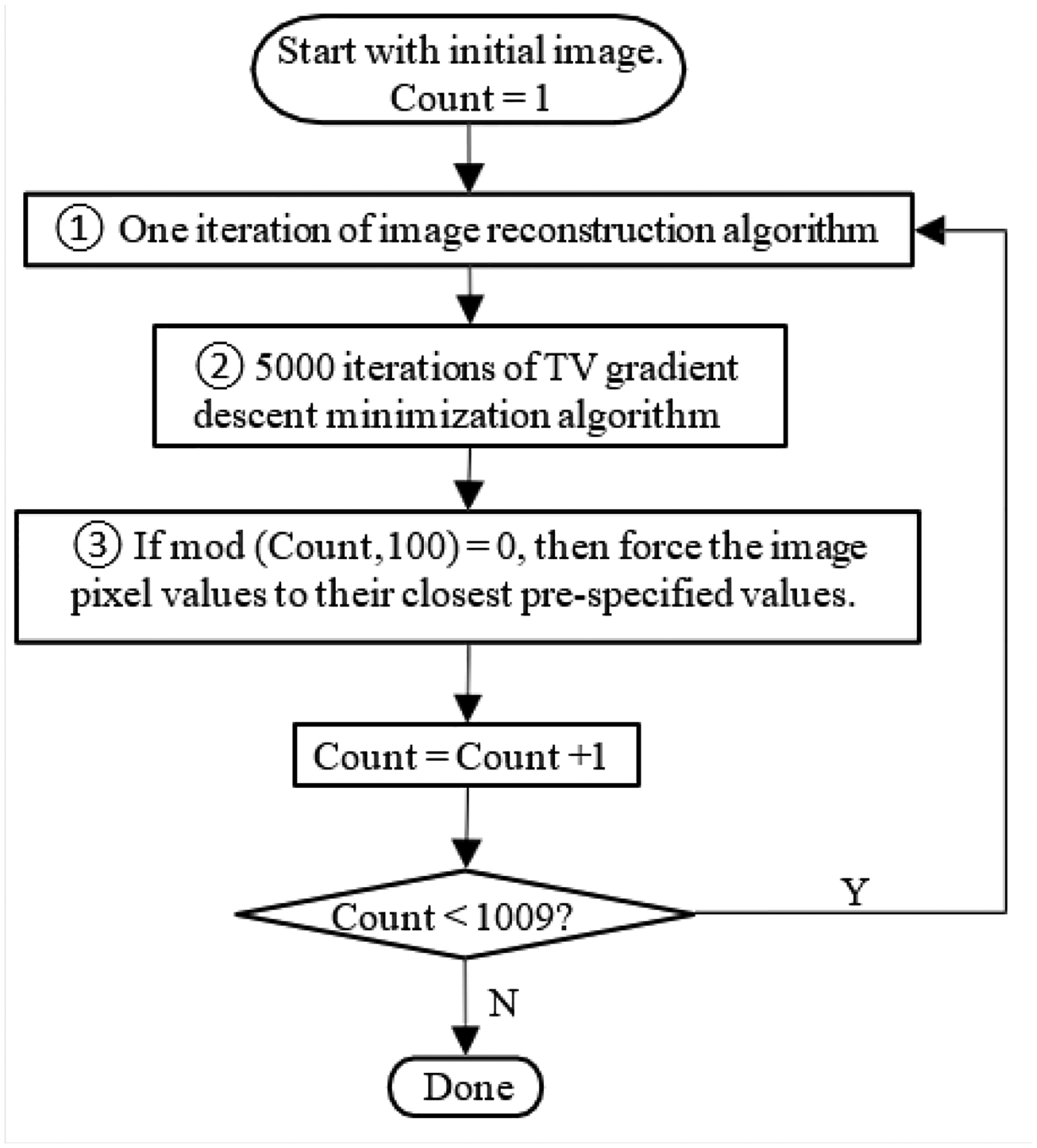
A flowchart of the algorithm used in computer simulations.

There are many approaches to develop an image reconstruction algorithm. One approach is to set up an objective function, which typically contains a data fidelity term and one or more Bayesian terms. Each Bayesian term represents a constraint. An algorithm that minimizes this objective function minimizes all the terms simultaneously. Another approach is the projections onto convex sets’ (POCS) approach. In this approach, the main algorithm consists of two or more sub-algorithms. These sub-algorithms work separately and sequentially. Each of them has its own goals in mind. For a POCS algorithm, it is not easy to study its convergence. However, it is easy to fine tune each sub- algorithm independently and to adjust the balance between them. The POCS approach is adopted in this paper and is described in Figure 1. The POCS algorithm we used in this paper consists of three sub-algorithms. The first sub-algorithm takes care of image reconstruction. Any iterative image reconstruction algorithm can potentially be used to minimize the discrepancy between the forward projection of the reconstructed image and the line- integral measurements. In Figure 1, the image reconstruction algorithm ① is chosen to be the well-known maximum- likelihood expectation-maximization (MLEM) algorithm [23]:

(1) $${x_{i,j}}^{\left(k+1\right)}-\frac{{x_{i,j}}^{\left(k\right)}}{\sum_{m}a_{i,j,m}}\sum_{m}a_{i,j,m}\frac{p_{m}}{\sum_{\hat{i},\hat{y}}a_{\hat{i},\hat{y},m}{x_{\hat{i},\hat{y}}}^{\left(k\right)}},$$

where *p*_*m*_ is the *mth* projection, *a*_*i, j,m*_, is the projection contribution from the pixel (*i*, *j*) to the projection bin *m*, and *k* is the iteration index. In fact, the user can choose any justifiable iterative image reconstruction algorithm for algorithm ①. For example, a transmission EM algorithm [24] or a least square minimization algorithm [5]. The second sub-algorithm is a gradient descent algorithm to minimize the TV norm of the reconstructed image. The gradient descent algorithm ② in Figure 1 is given as

(2) $${x_{i,j}}^{\left(k+1\right)}={x_{i,j}}^{\left(k\right)}-\eta \frac{\partial TV\left(X\right)}{\partial x_{i,j}},$$

where *η* = 2×10^−7^ bin our computer simulations, and is subdifferential of the TV norm of the current reconstructed image X. We use an extremely small step size *η* to ensure the stability of the algorithm. At the same time, we repeat this step 5000 times to guarantee the TV norm is effective. The TV norm can be defined as

(3) $$TV\left(X\right)=\sum_{i}\sum_{j}\sqrt{\left(x_{(i+1,j-x_{i,j}}\right)2+\left(x_{i,j+1}-x_{i,j}\right)2}.$$

One can combine algorithm ① and algorithm ② into one Bayesian algorithm [11].

The third sub-algorithm is used to enforce the reconstructed image pixels to take the pre-specified values. The sub-algorithm ③ is the new attempt in this paper. It simply moves its image pixel value to its closest default image values. For torso imaging, the default values can be set up as the linear attenuation coefficients of the air, soft tissues, and bones. In fact, this sub-algorithm is nothing but segmentation. Notice that this step is skipped for most iterations, as enumerated by the variable ‘Count.’ We only activate this step every 100 counts, as dictated by remainder function ‘mod’ in mod (Count,100) = 0, which is the reminder of Count/100. We only know the approximate potential values in the image. We must downplay these ‘known values’ constraint and give the overall POCS algorithm a chance to converge to the true values that may not be the same as our ‘set values.’ Therefore, it is important not to terminate the POCS with the sub-algorithm ③. The computer simulations in this paper consider a two- dimensional (2D) parallel-beam imaging system, with an image array size of 256 × 256 pixels, the detector size of 256 bins, and 8 views (over 180°). The projection line integrals were calculated analytically. The POCS algorithm used 1009 iterations used. Notice that 1009 is not a multiple of 100. This gives the pixel values in the reconstructed image a chance to move away from the segmented values set in the third sub-algorithm. Both noiseless data and noisy data were used in the computer simulations. The noise was Gaussian distributed with a mean value of 0 and a variance of 5.

## Results

**Figure 2:**
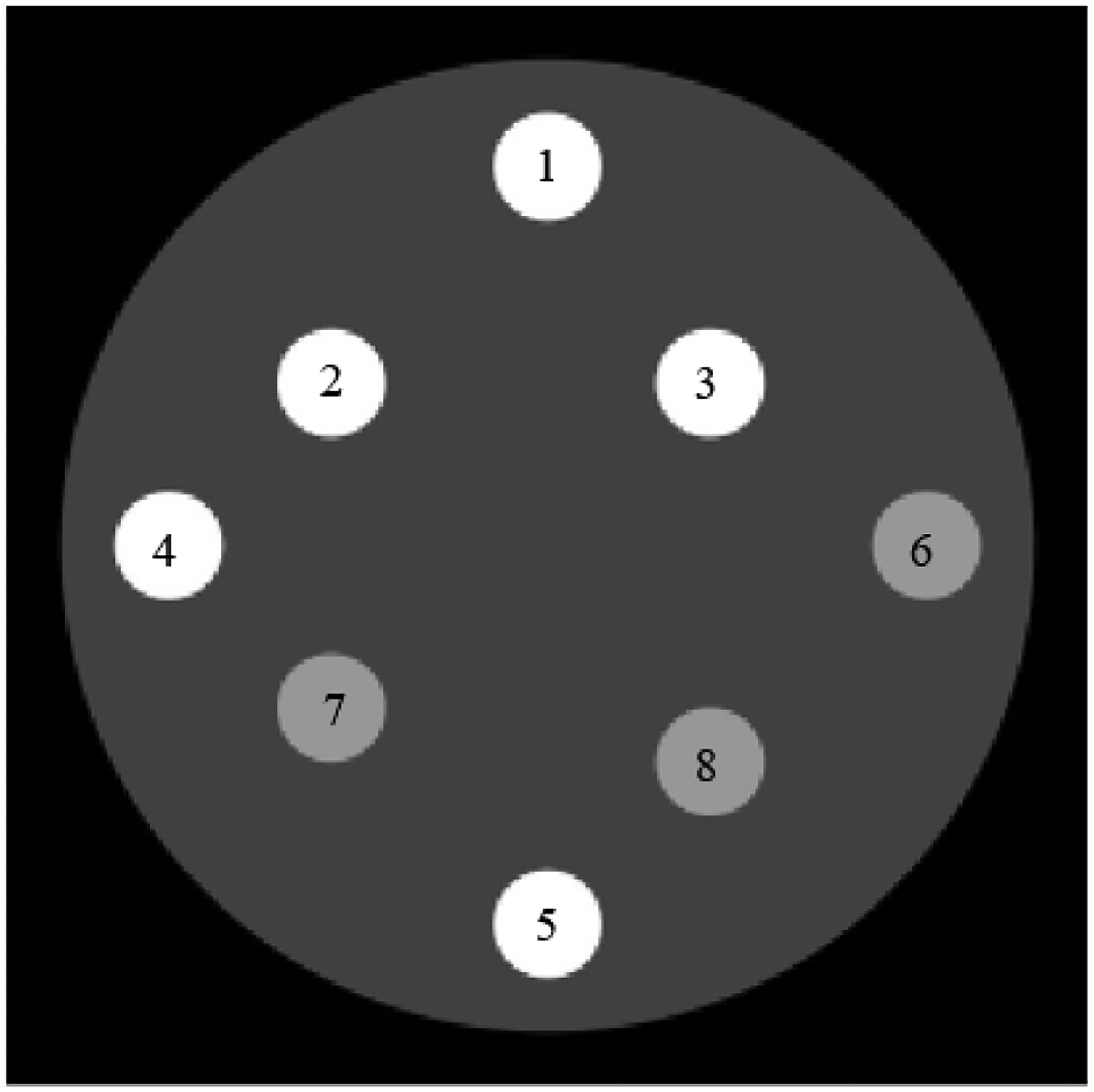
The true phantom.

Figure 2 shows the true phantom used in computer simulations. The large disk has a value of 0.5. There are 8 small discs. Discs 1–5 have a value of 1.5; discs 6–8 have a value of 1.0. In our implementation of the sub-algorithm ③, the potential pixel values were set at 0.51, 1.01, and 1.51. The corresponding pseudo code to update the pixel x (i, j) is as follows.

When ‘Count’ is a multiple of 100, execute.

if (0.25 < x(i, j) ≤ 0.75) then x(i, j) = 0.51;
if (0.75 < x(i, j) ≤ 1.25) x(i, j) = 1.01;
if (x(i, j) > 1.25) x(i, j) = 1.51.

**Figure 3:**
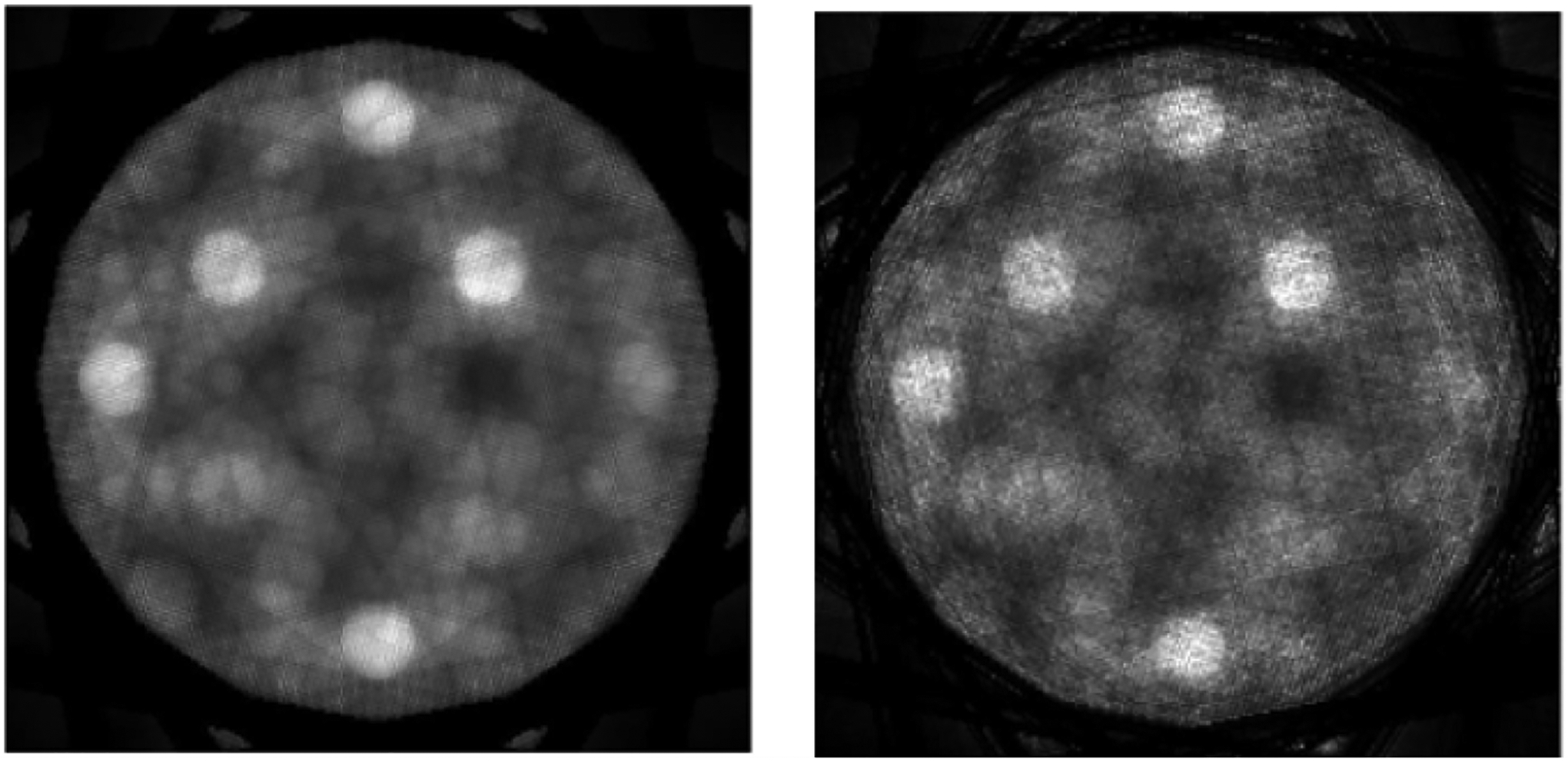
MLEM reconstructions: (Upper) using noiseless data; (Lower) using noisy data.

**Figure 4:**
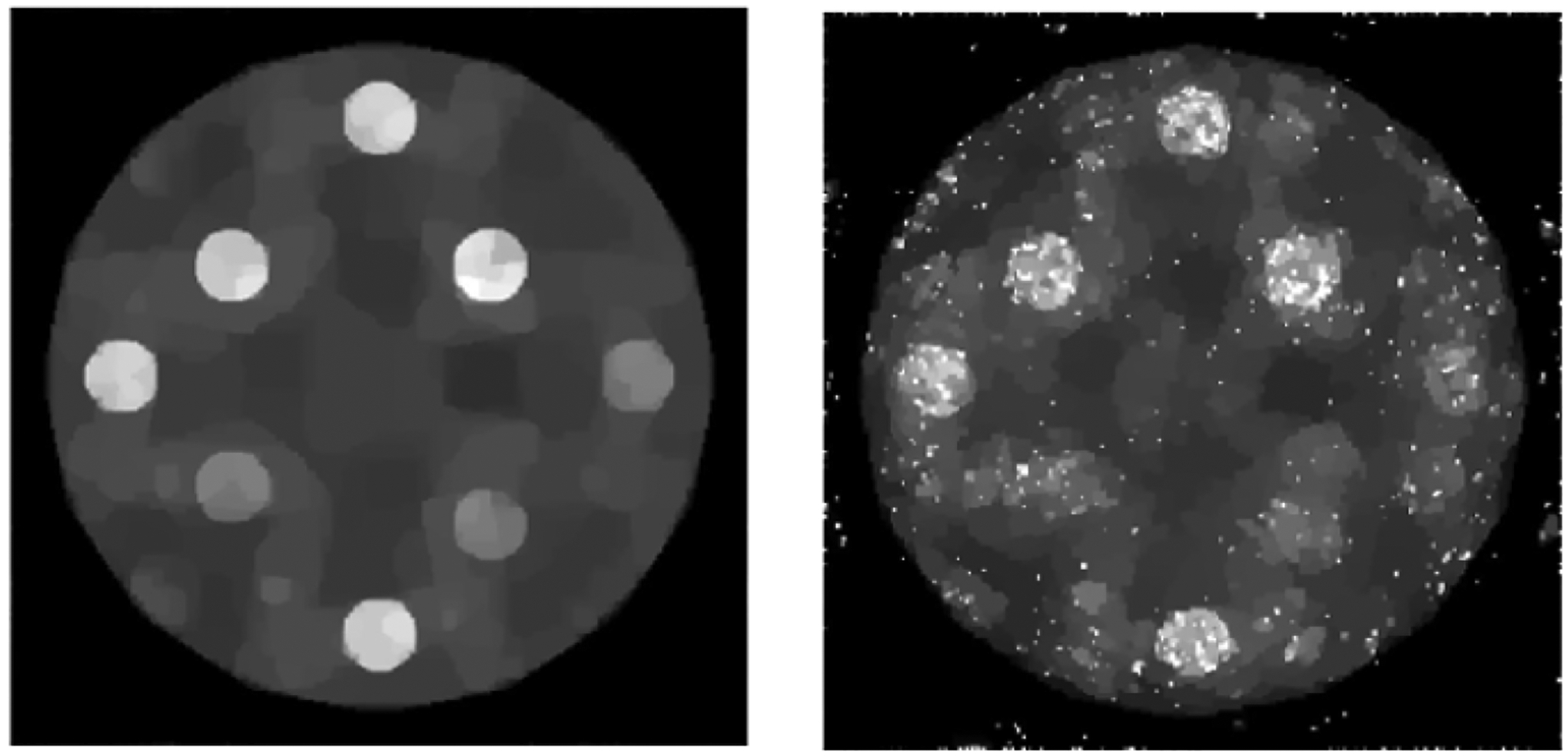
TV reconstructions: (Upper) using noiseless data; (Lower) using noisy data.

**Figure 5:**
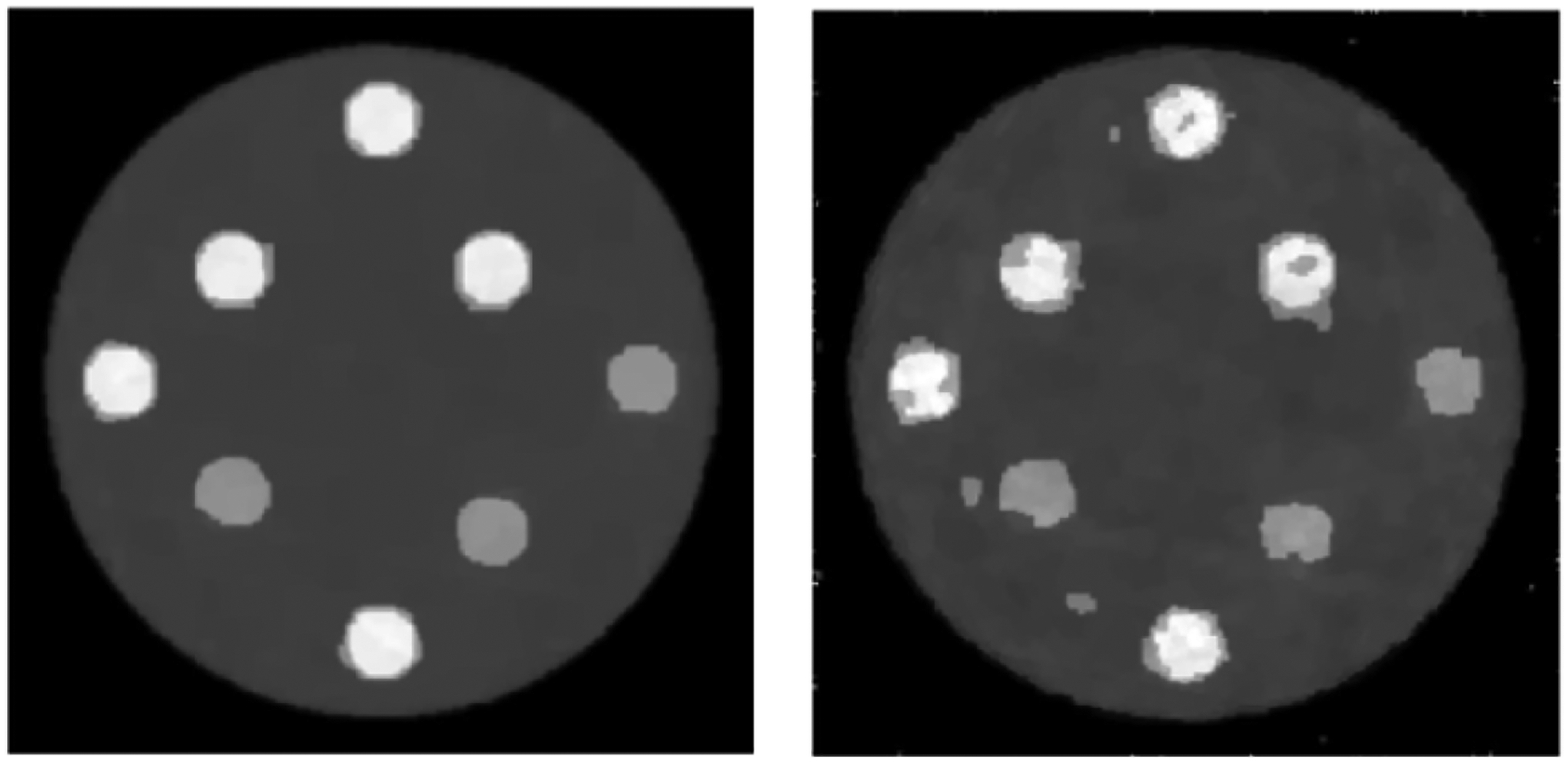
Proposed POCS reconstructions: (Upper) using noiseless data; (Lower) using noisy data.

We do not force any pixel to a hard zero in the sub-algorithm ③, because the MLEM algorithm cannot update the pixel value zero. Figure 3 shows two MLEM reconstructions, one with noiseless data and the other one with noisy data, respectively. Here, the sub-algorithms ② and ③ are disabled in Figures 1 & 2 shows two TV reconstructions, one with noiseless data and the other one with noisy data, respectively. Here, the sub- algorithm ③ is disabled in Figures 1 & 2 shows two proposed POCS reconstructions, one with noiseless data and the other one with noisy data, respectively. All images are displayed in the gray-scale window of [0, 1.59]. The structure similarity (SSIM), peak signal-to-noise ratio (PSNR), and signal-to-noise ratio (SNR) are compared for the reconstructions. Table 1 compares the results using the noiseless data; Table 2 compares the results using the noisy data. It is shown that the rough knowledge used in sub- algorithm ③ is helpful in obtaining better reconstructions (Figures 4 & 5).

## Discussion and Conclusion

One may ask; “What is the objective function of the sub- algorithm ③?” We do not need one. If one insists on having one, we can set something up as However, we do not suggest a gradient based algorithm to minimize (4). In our proposed POCS algorithm, we do not simply alternate between the sub-algorithms sequentially. Within each POCS iteration, we execute sub-algorithm ① once, sub-algorithm ② 5000 times, and sub-algorithm ③ 1/100 times. We run sub-algorithm ② 5000 times because we choose to use a very small step size in a gradient descent algorithm to minimize the TV norm. A larger step size produces worse images. A very small step size has almost no effect on the reconstructed image. To overcome this difficulty, we use a very small step size and a large iteration number for the sub- algorithm ②. As the sub-algorithm ③, the number of iterations is 1/100. In other words, this sub-algorithm is skipped 99 times for every 100 global POSC iterations. This is equivalent to re-setting the initial image every 100 global POCS iterations. One may argue that it is wrong to use the emission MLEM algorithm when the noise is Gaussian. The emission MLEM algorithm was originally derived for the Poisson noise. It is more proper to use a transmission EM algorithm or a least square error minimization algorithm for the sub-algorithm ①. Our attempt of using an emission MLEM algorithm is to demonstrate that the TV minimization makes the noise model less important. The Bayesian information dominates the noise model in the maximum likelihood. When the measurements are incomplete, any prior information and corresponding constraints will help. The piecewise feature of the objects makes the TV norm minimization effective. The rough knowledge of the image values, as demonstrated in this paper, is also effective. We believe that there are other features of the images that can be used to supplementing the incomplete data. Machine learning turns out to be an effective way to explore the common features for a group of similar images.

## Figures and Tables

**Table 1:** Comparison studies with noiseless data.

Method	SSIM	PSNR	SNR
Ideal case	1.00	∞	∞
MLEM w/o noise	0.4354	17.1834	11.2257
TV w/o noise	0.8692	23.1472	17.1895
Proposed POCS w/o noise	0.9472	26.4425	20.4847

**Table 2:** Comparison studies with noisy data.

Method	SSIM	PSNR	SNR
Ideal case	1.00	∞	∞
MLEM w/ noise	0.2545	15.9300	9.9723
TV w/ noise	0.5058	12.7521	6.7943
Proposed POCS w/ noise	0.8594	22.7771	16.8194

## Abbreviations:

TV: Total-Variation
POCS: Projections onto Convex Sets
PSNR: Peak Signal-To-Noise Ratio
SNR: Signal-To-Noise Ratio
MLEM: Maximum- Likelihood Expectation-Maximization
